# Difficulties in Treatment and Management of Epilepsy and Challenges in New Drug Development

**DOI:** 10.3390/ph3072090

**Published:** 2010-07-05

**Authors:** Abdul Wahab

**Affiliations:** Institute of Neurophysiology, Charité Berlin Medical University, Tucholskystrasse 2, D-10117 Berlin, Germany; E-Mail: abdul.wahab@charite.de; Tel.: +49 30 44707274; Fax: +49 30 450528962

**Keywords:** antiepileptic, anticonvulsant, seizure, pharmacoresistance, refractory seizures

## Abstract

Epilepsy is a serious neurological disorder that affects around 50 million people worldwide. Almost 30% of epileptic patients suffer from pharmacoresistance, which is associated with social isolation, dependent behaviour, low marriage rates, unemployment, psychological issues and reduced quality of life. Currently available antiepileptic drugs have a limited efficacy, and their negative properties limit their use and cause difficulties in patient management. Antiepileptic drugs can provide only symptomatic relief as these drugs suppress seizures but do not have ability to cure epileptogenesis. The long term use of antiepileptic drugs is limited due to their adverse effects, withdrawal symptoms, deleterious interactions with other drugs and economic burden, especially in developing countries. Furthermore, some of the available antiepileptic drugs may even potentiate certain type of seizures. Several *in vivo* and *in vitro* animal models have been proposed and many new antiepileptic drugs have been marketed recently, but large numbers of patients are still pharmacoresistant. This review will highlight the difficulties in treatment and management of epilepsy and the limitations of available antiepileptic drugs and animal seizure models.

## 1. Introduction

Epilepsy is perhaps one of the oldest recorded medical illnesses in history. In ancient times, epilepsy was described as a condition representing an evil state of mind or possession, while on the other hand. in some cultures patients with epilepsy were regarded as people who had mystical or spiritual powers, and it was even thought to be contagious [1,2]. Hippocrates linked the seizures to a problem in brain function more than 2,000 years ago. The first modern definition of epilepsy was given in 1875 by Hughling Jackson, who recognized a seizure as being due to disordered brain electricity, which can alter consciousness, sensation, and behavior [3]. The discovery of the EEG in the 1920s helped in correlating the neuronal activities to the behavioral disorders [4]. Currently, advances in medical imaging techniques, as well as in genetics, have resulted in new and improved treatment approaches [5,6,7]. During the last two decades, several new antiepileptic drugs and improved formulations of older drugs have been licensed for the treatment of epilepsy [8,9,10,11]. Several *in vivo* and *in vitro* models have been proposed for identifying the compounds for the treatment of epilepsy [12,13,14,15]. Despite these advances, the cellular basis of epilepsy is still a mystery and around 30% of epileptic patients are pharmacoresistant and the seizures and medications are still the major reasons of morbidity and mortality of epileptic patients.

## 2. Epidemiology of Epilepsy

Epilepsy is one of the most common serious neurological disorders, responsible for substantial morbidity and mortality due to the seizures and the available medications. Around 50 million people in the world have epilepsy and approximately 5% of the general population experience at least one seizure, excluding febrile seizures, at some time in their lives [16,17,18]. The prevalence of epilepsy is around 0.5-1% [19], and its overall annual incidence ranges from 50-70 cases per 100,000 in industrialized countries and up to 190 per 100,000 in developing countries [16,20]. Around 80% of people with epilepsy reside in developing countries [20,21,22,23]. The high incidence in developing countries is attributed to poor obstetric services and the greater risk of intracranial infections and head injuries [24]. Furthermore in these countries 80-90% of epileptic patients have difficulties in accessing treatment [20,21,22]. This treatment gap has been mainly ascribed to inefficient and unevenly distributed health-care systems, cost of treatment, cultural beliefs, and unavailability of antiepileptic drugs [22,23]. The diagnosis of epilepsy in developing countries is a difficult task. Video-electroencephalogram (EEG) and ambulatory long-term EEG monitoring provide a great help for the differential diagnosis of epilepsy and other paroxysmal events, but in many areas of developing countries, especially rural areas, these diagnostic techniques are not available. Due to lack of these facilities, high rates of misdiagnosis are likely in these countries. Most studies suggest there is a slightly higher incidence of epilepsy in males then females [17,18].

Mortality in epileptic patients is two- to three-times that of the general population, and sudden unexpected death in epilepsy (SUDEP) is the most important direct epilepsy-related cause of death [25,26,27]. SUDEP is defined as a non-traumatic and non-drowning death in patients with epilepsy that is sudden, unexpected, witnessed or unwitnessed, and with or without evidence of a seizure [25]. It occurs in 1-3 per 1,100 epileptic patients per year [28]. Risks for SUDEP are higher if patients are male, 20-40 years of age, have generalized seizures, and are pharmacoresistant [26,28,29,30]. 

## 3. Age Groups

The incidence of epilepsy is higher in childhood and in the elderly than in young people [18,31]. In about 50% of cases the onset of epilepsy occurs in childhood and in the elderly, with half of those being under one year of age [19]. It has been well established that early in life the brain is more seizure susceptible, and seizures in the immature brain are likely to be dependent on different mechanisms than those in the adult [32,33]. Epilepsies in early childhood frequently are difficult to treat. This may depend on physiological immaturities in ion homeostasis and other developmental characteristics, but also on the severity of early onset epilepsy. Neonatal brain dysfunction and its behavioral expression may originate in the antepartum period [34,35]. It seems that short gestational age, low birth weight, and intrauterine growth restriction are associated with an increased risk of afebrile seizures in the first year of life [36]. Children of a low birth weight or born preterm also have an increased risk of febrile seizure [37]. Early-life seizures do induce much less chronic morphologic changes in the hippocampus than seizures in adult temporal lobe epilepsy [38,39,40,41,42,43,44]. Recurrent early life seizures may nevertheless result in permanent behavioural abnormalities and enhance epileptogenicity [45]. 

Epileptic seizures are considered as the third most frequent neurological problem encountered in the elderly [46]. Treatment of epilepsy in the elderly is complicated since these patients are very often prescribed other long term medication for disorders other than epilepsy that may result in deleterious drug interactions [46,47]. 

## 4. Pharmacoresistant Epilepsy

Pharmacoresistance may be defined as poor seizure control despite accurate diagnosis and carefully monitored pharmacologic treatment. Clinically available anticonvulsant drugs fail to control seizures in around 30% of epileptic patients, which constitutes some 15 million people in the world [48,49]. About 75% of patients diagnosed with mesial temporal lobe epilepsy have pharmacoresistant seizures [50,51] and more than 50% of patients with Lennox–Gastaut syndrome are classified as pharmacoresistant [52]. The condition is more complicated in certain brain abnormalities, for example, when hippocampal sclerosis is combined with focal dysplasia or similar developmental alterations the chances of pharmacoresistance may reach more than 90% [53]. In most of the patients antiepileptic drugs are prescribed for lifelong treatment as patients who become seizure free with the use of antiepileptic drugs have a high likelihood of relapse following the discontinuation of medication [54,55] and early treatment does not influence the probability of long-term remission [56]. Despite the introduction of new drugs, the problem of pharmacoresistance has not been solved, although most of the new drugs have better safety profiles than those of older drugs. Surgical treatment of epilepsy may be an alternative, but at present, surgery is possible in only a small proportion of pharmacoresistant patients and after the surgery most of the patients are still prescribed antiepileptic drugs for full seizure control, thus this cohort of patients is still at risk for deleterious side effects and drug interactions. Continuous therapy with antiepileptic drugs cannot prevent the development of pharmacoresistance as it has been reported that some patients who become seizure free initially with the use of antiepileptic drugs develop pharmacoresistance later [57]. Moreover, pharmacoresistance is associated with social isolation, dependent behaviour, low rates of marriage, unemployment, psychological issues, medical comorbidities due to medication and seizures, reduced quality of life, and an economic burden on society [58,59,60]. It is not well established why and how epilepsy becomes drug resistant in some patients while others with seemingly identical seizure types and epilepsy syndromes can achieve seizure control with medication. Thus, there is a clear need to understand the pathomechanisms involving epilepsy and new drugs with improved efficacy and safety profile for pharmacoresistant patients. 

Three major pathomechanisms have been proposed to explain pharmacoresistance in around 30% of patients: disease-related mechanisms, genetics and drug-related mechanisms [61,62]. Two key hypotheses have been proposed as disease-related mechanisms. The target hypothesis proposes the alterations of pharmacological targets of antiepileptic drugs in the brains of pharmacoresistant patients that lead to the failure of antiepileptic drugs to block excitatory sodium or calcium currents or to enhance the GABA-mediated inhibition, whereas the transporter hypothesis proposes that excessive expression of multidrug transporters could remove antiepileptic drugs from epileptogenic brain regions [63]. Genetic alterations due to, for example, polymorphisms in drug efflux transporters may also lead to poor seizure control in these patients [63]. Finally, tolerance as a drug-related mechanism may be responsible for lower efficacy of antiepileptic drugs in these patients. Intensive research is being carried out based on these hypotheses; however the detailed mechanisms leading to pharmacoresistance is still unknown.

## 5. History of Antiepileptic Drug Development

Epileptic disorders have been treated for thousands of years with a variety of botanicals and herbs [64,65]. Potassium bromide was the first single compound, which was used for the treatment of epilepsy following the serendipitous (chance) discovery of its activity in this area by Sir Charles Locock in 1857 [66,67]. Because bromide salts were the only drug available for the treatment of epilepsy at that time, these were used regularly for the next 50 years, despite their limited efficacy and terrible side effects. Phenobarbital was the second single compound discovered serendipitously for the treatment of epilepsy. Its anticonvulsant properties were accidentally discovered in 1912 by Alfred Hauptmann, who originally used it as a tranquilizer for his epileptic patients and found that phenobarbital attenuated the epileptic attacks of these patients [68]. Since that time phenobarbital has been widely used as an antiepileptic drug worldwide [67]. The use of a ketogenic diet was introduced for the treatment of epilepsy in the 1920s [69,70,71,72]. This special diet, which has high fat, low protein, and negligible amounts of carbohydrate content, was prepared to mimic some of the characteristics of fasting, a state known to decrease seizures in some individuals [73]. Phenytoin, one of the drugs of first choice for generalized tonic clonic and partial seizures, was the first antiepileptic drug discovered using an animal seizure model. Phenytoin was synthesized in 1908, and was recognized as a first non sedating antiepileptic drug after the pioneering studies of Merritt and Putnam using an electroshock-induced seizure model in cats [74,75,76]. Since then electroshock-induced seizure model has been used for identifying the new compounds for the treatment of epilepsy and drugs that are effective in blocking tonic hind limb extension in animals induced by electroshock generally have been found to be effective against generalized tonic clonic seizures in human beings [14,15]. 

Trimethadione, the first treatment specifically for absence seizures was licensed in the 1940s, following laboratory evaluation with the pentylenetetrazole animal seizure model by Richards and Everett in 1944 [77] and clinical evaluation by Lennox in 1945 [78]. Primidone was introduced as an antiepileptic drug in the 1950s. It is metabolized into the active compounds phenobarbital and phenylethylmalonamide, but because it is associated with higher incidences of side effects, therefore other drugs are preferred over primidone for clinical use [66,79]. Ethosuximide was introduced into clinical practice in 1960 and has been the drug of choice for children with absence seizures [80]. Diazepam, widely used for the treatment of status epilepticus, was introduced in the late 1960s [81]. Carbamazepine was synthesized by Schindler in 1953 [82] and was first marketed as a drug to treat trigeminal neuralgia in the 1960s. Later its antiepileptic effect was discovered and it was marketed as an antiepileptic drug in the 1970s [66]. Valproic acid was marketed in the late 1970s following a serendipitous discovery in 1963, when it was used as a solvent for a number of other compounds that were being screened for anticonvulsant activity [83]. 

During the last two decades, several new drugs have been licensed for the treatment of epilepsy including felbamate, gabapentin, lamotrigine, topiramate, tiagabine, levetiracetam, oxcarbazepine, zonisamide, pregabalin, rufinamide, stiripentol, clobazam, vigabatrin, and lacosamide [9,10,11,84]. In addition, new formulations of older drugs have also been marketed including fosphenytoin, a pro-drug of phenytoin, and a sustained-release preparation of carbamazepine. Furthermore, vagus nerve stimulation in combination with seizure medication has been licensed for partial epilepsy in adults [8,85,86]. In current times research is not only focused on chemical compounds, but also on implantable antiepileptic devices, which are under investigation [86].

## 6. Limitations of Antiepileptic Drug Therapy

Currently available antiepileptic drugs have limited efficacy, and their negative properties limit their use and cause difficulties in patient management. Antiepileptic drugs can provide only symptomatic relief as these drugs suppress seizures and have no effect on the epileptogenesis, which is a process that converts the normal circuitry of the brain into a hyperexcitable one, often after an injury [87,88,89,90]. The long term use of antiepileptic drugs is limited due to their adverse effects, withdrawal symptoms, deleterious interactions with other drugs and economic burden, especially in developing countries [91,92,93]. 

Despite huge funding, and extensive premarketing testing for the adverse effects of new antiepileptic drugs, they may still show severe side effects after being introduced on to the market [93]. For example, unexpected visual field defects have been observed in patients taking vigabatrin a few years after its introduction to the market [94,95] and in a high number of individuals felbamate unexpectedly caused aplastic anemia and hepatitis, which were not observed during clinical trials with this drug [8,96]. The trials which are carried out before marketing new antiepileptic drug typically involve a limited number of patients, so very rare but severe side effects may go unnoticed during these trials. Furthermore, most premarketing clinical trials in pharmacoresistant patients with new antiepileptic drug are not of considerably long duration, whereas for chronic epileptic patients antiepileptic drugs are prescribed for several years and may be lifelong, therefore it is possible that long term adverse effects go undetected during the trials [93]. 

The older antiepileptic drugs exhibit more serious side effects than newer antiepileptic drugs (Table 1). The main limitation of phenobarbital is its tendency to alter cognition, mood, and behavior [97]. 

**Table 1:** Clinical uses in common seizure types and syndromes and adverse effects of antiepileptic drugs.

Drugs	Clinical uses	Adverse effects
Phenobarbital	Partial and generalized seizures (ineffective against absence seizures), status epilepticus	Sedation, lethargy, dysarthria, coarsening of facial features, skin rashes, Dupuytrens’ contracture, reduced libido, osteomalacia, cognitive problems, insomnia (in children), distractability (in children), hyperkinesia (in children), irritability (in children), hepatoxicity, teratogenicity
Phenytoin	Partial seizures, generalized tonic-clonic seizures, status epilepticus, (ineffective against absence and myoclonic seizures)	Ataxia, diplopia, nystagmus, coarsening of facial features gingival hyperplasia, hirsutism, skin rashes, Stevens–Johnson syndrome, Dupuytren’s contracture, agranulocytosis, aplastic anemia, hepatoxicity, teratogenicity
Ethosuximide	Absence seizures	Gastrointestinal changes, drowsiness, lethargy, mood changes, headache, visual changes, aplastic anemia, agranulocytosis
Carbamazepine	Partial seizures, generalized tonic-clonic seizures, (ineffective against absence and myoclonic seizures)	Diplopia, dizziness, headache, ataxia, nystagmus, skin rashes, hyponatremia, aplastic anemia, agranulocytosis, weight gain, Stevens–Johnson syndrome, osteomalacia, hepatoxicity, teratogenicity
Benzodiazepines	Status epilepticus, partial and generalized seizures	Sedation, lethargy, drowsiness, dizziness, behavioral disturbances in children, hypersalivation
Valproic acid	Partial and generalized seizures	Tremor, weight gain, dyspepsia, diarrhea, peripheral edema, pancreatitis, hair loss, thrombocytopenia, agranulocytosis, polycystic ovaries, Stevens–Johnson syndrome, hepatoxicity, teratogenicity
Felbamate	Severe and/or refractory epilepsies including Lennox-Gastaut syndrome	Anorexia, weight loss, insomnia, dizziness, headache, ataxia, skin rashes, aplastic anemia, hepatoxicity
Gabapentin	Adjunct for partial seizures (ineffective against absence and myoclonic seizures)	Drowsiness, dizziness, ataxia, fatigue, hyperactivity (in children), weight gain
Pregabalin	Adjunct for partial seizures	Weight gain, peripheral edema, dizziness, somnolence, asthenia, headache, ataxia
Lamotrigine	Adjunct for partial and generalized seizures (may aggravate severe myoclonic	Dizziness, sedation, headache, diplopia, ataxia, skin rash, Stevens-Johnson syndrome
	epilepsy of infancy), Lennox-Gastaut Syndrome	
Topiramate	Adjunct for partial and generalized Seizures	Cognitive problems, word finding difficulty, kidney stones, paresthesias, anorexia, weight loss, acute angle closure glaucoma
Tiagabine	Adjunct for partial seizures (ineffective against absence	Dizziness, lethargy, tremor, nervousness, emotional changes
	seizures)	
Vigabatrin	Infantile spasms, refractory partial seizures (ineffective against absence and myoclonic seizures)	Drowsiness, dizziness, ataxia, tremor, lethargy, insomnia, Irritability and hyperactivity (in children), psychosis and depression, weight gain, visual field defects and blindness
Oxcarbazepine	Partial seizures, generalized tonic-clonic seizures (ineffective against absence and myoclonic seizures)	Drowsiness, dizziness, diplopia, headache, fatigue, GI distress, hyponatremia, skin rash, Stevens-Johnson syndrome
Zonisamide	Partial and generalized seizures	Sedation, dizziness, headache, GI distress, skin rash, aplastic anemia, agranulocytosis, kidney stones, weight loss
Levetiracetam	Adjunct for partial and generalized tonic-clonic seizures	Sedation, fatigue, dizziness, headache, anorexia, psychiatric disturbances, leucopenia
Lacosamide	Adjunct for partial seizures	Dizziness, nausea, diplopia, blurred vision, vomiting, headache, tremor and somnolence
Rufinamide	Lennox-Gastaut syndrome	Somnolence, vomiting, pyrexia, diarrhea and rash
Stiripentol	Dravet’s syndrome	Loss of appetite, drowsiness, cognitive impairment, ataxia, diplopia, nausea, abdominal pain

References: [8,79,80,90,92,97,98,100,105,178].

With phenytoin and carbamazepine treatment vestibulocerebellar symptoms, such as ataxia, diplopia, nystagmus, and vertigo, are common [79,80]. Ethosuximide most commonly causes gastro-intestinal symptoms, drowsiness, and headache. Allergic rashes occur in approximately 5% of patients with the use of ethosuximide [98]. Tachyphylaxis is associated with the use of benzodiazepines [99,100]. Hepatoxicity may result with the use of valproic acid, felbamate, carbamazepine, phenytoin and phenobarbital [101,102]. Weight gain is reported with the use of gabapentin, pregabalin, valproic acid, vigabatrin and possibly carbamazepine, whereas weight loss is reported with the use of felbamate, topiramate, and zonisamide [103,104]. Patients taking phenytoin, carbamazepine, phenobarbital, primidone, oxcarbazepine, zonisamide and particularly lamotrigine are at high risk of developing skin rashes [105,106,107]. Topiramate has been reported to have deleterious effects on cognition [108]. Hyponatremia is associated with the use of carbamazepine and oxcarbazepine [109]. The use of topiramate and zonisamide has been associated with increased risk of kidney stones [96,98]. Antiepileptic drugs prescribed to pregnant women may cause fetal malformations including neural tube defects, cognitive impairment, congenital heart defects, orofacial clefts, minor anomalies, growth retardation, developmental delay and microcephaly [110,111,112,113,114,115,116]. Furthermore, some of the available antiepileptic drugs may even potentiate certain types of seizures for example carbamazepine and vigabatrin have been reported to precipitate or aggravate absence, myoclonic, and complex partial seizures [92,117,118]. Gabapentin has been reported to induce absence and myoclonic seizures [92,108,119]. Aggravation of myoclonic, tonic-clonic, and absence seizures have also been documented with the use of ethosuximide [117]. Benzodiazepines occasionally have been reported to precipitate tonic seizures, especially when given intravenously to control other seizure types in patients with Lennox-Gastaut syndrome [119]. Phenobarbital, lamotrigine, and phenytoin have been reported to induce absence, myoclonic and complex partial seizures respectively [90,92,118,120]. 

Treatment with two or more drugs (polytherapy) may results in drug-drug interactions that may increase the chances of antiepileptic drug toxicity. Pharmacoresistant patients often require treatment with one or more antiepileptic drugs. To further complicate matters most elderly epileptic patients are often prescribed other medications in addition to antiepileptic drugs. Some antiepileptic drugs induce hepatic metabolizing enzymes, e.g. phenytoin, carbamazepine, phenobarbital, and primidone, whereas others inhibit these enzymes, e.g. valproic acid [121]. Drug interaction may also occur for competition for drug binding sites in plasma. Therefore, it has been suggested that before long-term treatment with polytherapy, all reasonable options for monotherapy should be exhausted [97]. For polytherapy, antiepileptic drugs not metabolized by the liver and those with no or low protein binding should be preferred. 

Noncompliance in patients with epilepsy is a serious barrier to successful treatment and a major source for seizure breakthrough [80,122]. Noncompliance is perhaps the single most important factor in increasing the costs of care for people with epilepsy. The major reasons of non compliance are inconvenient doses, complicated regimens, and side effects. Monitoring the dosing in newly treated and long-term patients using a computerized pill box, has revealed that no patients were completely compliant with their medications, and the noncompliance of just a short duration was often linked with the occurrence of breakthrough seizures, despite many months of successful treatment [123]. Higher rates of compliance has been found with once or twice a day dosing whereas poor compliance has been found with drugs, which require 3 or 4 times dosing in a day [124]. Therapeutic drug concentration monitoring in serum may be helpful in detecting or confirming poor compliance and studying the interindividual variation in pharmacokinetics and the factors responsible for such variation [125].

Although the new antiepileptic drugs do not offer significant advantages in terms of efficacy compared to the older ones, they do offer some benefits in terms of tolerability, fewer drug interactions and simpler pharmacokinetics [15,126]. Higher rates of compliance can be achieved with new antiepileptic drugs since most of them have long half-lives, and can be administered once or twice daily. Possibilities of drug interactions are less with newer antiepileptic drugs since most of them are minimally or not bound to plasma protein [11,127]. Enzyme-inducing properties, which is the great source of drug interaction among old antiepileptic drugs, is absent in some of the new antiepileptic drugs [11] Furthermore, animal studies have indicated less teratogenetic potentials for most of the new antiepileptic drugs [127]. 

## 7. Economic Failure of Antiepileptic Drugs

The development of new drugs is costly and risky. The chances for successful completion of development and approval by the regulatory authorities are less than 10%, even for those drugs which are in Phase 1a stage [10,128]. Despite huge funding for new antiepileptic drug development, the drugs lack safety and efficacy, and historically many antiepileptic drugs were withdrawn from the market because of their severe adverse effects. 

For instance, phethenylate was withdrawn because it was producing hepatic necrosis, benzchlor-propamide demonstrated toxic effects with long-term use in animals, and aminoglutethimide was linked to the high incidence of goiter [81]. The use of felbamate has been restricted because of its potential to cause aplastic anemia and hepatic failure, discovered after its marketing, and similarly, use of vigabatrin is not so common because it was found to cause visual field defects [94,100,108,129,130]. Furthermore newly developed antiepileptic drugs are expensive and their lifelong use makes it almost impossible to afford for the people of third world countries. However, the cost of antiepileptic drug development is compensated because of their possible use in other disorders (Table 2). The research on compounds derived from medicinal plants may provide the safe drug if they are already consumed by people. 

**Table 2:** Uses of antiepileptic drugs in non-epilepsy disorders.

**Drugs**	**Clinical uses in non-epilepsy disorders**
Phenobarbital	Insomnia*, essential tremor
Benzodiazepines	Anxiety, insomnia, essential tremor, restless legs syndrome, alcohol withdrawal, [neuropathic pain]
Carbamazepine	Trigeminal neuralgia, neuropathic pain, bipolar disorder, alcohol withdrawal
Valproic acid	Migraine prophylaxis, bipolar disorder, [neuropathic pain]
Gabapentin	Neuropathic pain e.g. postherpetic neuralgia, migraine prophylaxis, anxiety, Restless legs syndrome, [essential tremor]
Lamotrigine	Bipolar depression, neuropathic pain, trigeminal neuralgia, [migraine prophylaxis]
Pregabalin	Neuropathic pain, anxiety
Tiagabine	[Neuropathic pain, anxiety, migraine prophylaxis]
Topiramate	Migraine prophylaxis, essential tremor, [neuropathic pain, anxiety, bipolar disorder]
Oxcarbazepine	Neuropathic pain, bipolar disorder, trigeminal neuralgia
Levetiracetam	Neuropathic pain, [migraine prophylaxis, essential tremor]

* Indications shown without parentheses are widely accepted, and indications shown in parentheses are preliminary. References [179,180,181,182,183,184,185,186,187].

## 8. Animal Seizure Models

All antiepileptic drugs are tested extensively in animal models for efficacy and safety before their use in humans, as animal seizure models provide some knowledge of the possible clinical use of new compounds. For example, it is predicted that the compounds which show efficacy in the maximal electroshock seizure test may be used for the treatment of generalized tonic clonic seizures in humans and compounds which show efficacy in the pentylenetetrazole model may be used for the treatment of myoclonic seizures in human patients [14,131]. Animal seizure models are developed by using chemical or electrical stimulus-evoked paradigms to generate seizure activity *in vivo* or *in vitro*. 

Animal seizure models have widely been used after the successful discovery of antiepileptic drugs phenytoin using the electroshock seizure test and trimethadione using the pentylenetetrazole test [74,77,132]. Both the electroshock seizure test and the pentylenetetrazole seizure test are still used for screening purposes [133,134,135,136]. It is assumed that the pentylenetetrazole seizure model may be a good predictor of clinical efficacy against absence seizures. But this may not necessarily be the case, for example, phenobarbital, primidone, gabapentin and tiagabine block pentylenetetrazole induced seizures but are not effective against absence seizures in human patients and they may even exacerbate absence seizures in human patients [135]. It should also be noted that lamotrigine was found ineffective against the pentylenetetrazole model [137], but has demonstrated some efficacy against absence seizures in humans [138,139]. It has been argued that the use of these models will detect the antiepileptic drug similar to those which are already in market and therefore the potentially useful compounds may be missed using these models. For example, the maximal electroshock seizure and pentylenetetrazole tests failed to identify the anticonvulsant activity of levetiracetam. However, levetiracetam was subsequently found to be active in other seizure models [140,141,142]. Therefore, it is suggested that along with these models, other seizure models should also be used for development of new antiepileptic drug since no single test can satisfactorily demonstrate the anticonvulsive properties of new compounds. 

*In vitro* models in this regard are very important although they will not be able to predict the pharmacokinetic profile of a new compound but they can detect the anticonvulsant properties of even those compounds which have poor pharmacokinetic profiles. So if such compounds are found to be active using *in vitro* models, one can modify the pharmacokinetic profile of these compounds by making derivatives. This strategy should help us to get a compound with novel structure and unique profile. A number of *in vitro* seizure models are available [12,53]. In entorhinal-hippocampal acute slices prepared from adult rats, seizure like events can be induced by elevation of K^+ ^ [143], lowering of Ca^2+^ [144], or lowering of Mg^2+^[145] in artificial cerebrospinal fluid (ACSF). In addition, interference with ion channels can also induce such kind of activities. Therefore, application of 4-aminopyridine, which is well known to interfere with different types of K^+^ channels, induces seizure-like events in acute hippocampal slices [146,147]. *In vitro* models of epileptiform activities have also been developed using electrical stimulation [148]. Seizure-like afterdischarges can be induced by high-frequency electrical stimulation to the stratum radiatum of the CA1 region of rat hippocampal acute slices and organotypic hippocampal slice cultures [149,150,151]. All of the above mentioned *in vitro* seizure models respond to most of the clinically used antiepileptic drugs and can be used for screening purposes along with *in vivo* seizure models. 

Several *in vivo* and *in vitro* models have been proposed as models of pharmacoresistance on the basis that the seizures do not completely respond to current antiepileptic drugs. The *in vivo* pharmacoresistant models include the phenytoin-resistant kindled rat [152] the lamotrigine-resistant kindled rat [153] the 6 Hz psychomotor seizure model of partial epilepsy [154,155], poststatus epileptic models of temporal lobe epilepsy [156,157,158] and the methylazoxymethanol acetate *in utero* model of nodular heterotopias [159]. The *in vitro* pharmacoresistant models have been developed using acute hippocampal entorhinal cortex slices prepared from adult rats. In these slices combined application of bicuculline and 4-aminopyridine induces pharmacoresistant recurrent discharges [160]. In these acute slices, the seizure like events appearing in the entorhinal cortex during initial stages of low Mg^2+^ ACSF perfusion are pharmacosensitive, whereas late recurrent discharges that appeared in the entorhinal cortex during later stages of low Mg^2+^ ACSF perfusion are pharmacoresistant [161,162]. *In vitro* preparations from immature tissue have been found more pharmacoresistant than those from adult tissue [33]. Seizure like events induced in organotypic hippocampal slice cultures prepared from immature tissue have been found to be pharmacoresistant, the seizure like events can be induced even in two month-old slice cultures [163,164]. Pharmacoresistance of seizure like events has also been found in acutely prepared intact hippocampus from immature rats [165]. *In vitro* recordings from human brain slices prepared after surgery of pharmacoresistant epileptic patients is also a valuable tool to investigate mechanisms of pharmacoresistance [166,167,168,169,170]. 

In addition to these *in vivo* and *in vitro* seizure models, other models are also available. For example, the γ-hydroxybutyrate spike-and-wave model of absence seizures shows a cascade of EEG and behavioral events similar to human absence seizures and is used to evaluate new compounds which may be used for the treatment of human generalized absence seizures [13,15,171]. Standard antiepileptic drugs like valproic acid and ethosuximide, which are used for the treatment of human absence seizures, are effective in this model, whereas phenytoin and carbamazepine, which are ineffective in human absence seizures, enhanced the γ-hydroxybutyrate induced spike–wave bursts. 

The kindling model is a chronic model in which recurrent electrical or chemical stimulations is used, usually daily, until the animal develops generalized convulsions in response to the stimulus [172]. This model is useful for identifying activity against partial seizures with a secondary generalization [14,173]. A compound can be administered during the whole kindling procedure or prior to a kindling stimulus in an animal previously fully kindled. The limitation of this model is that it is extremely labor intensive and difficult to be used for screening purposes.

The 6 Hz psychomotor seizure test has been proposed as a model of partial epilepsy. In this test mice are subjected to low-frequency (6 Hz), long duration (3 s) stimulation that produces seizures similar to those observed in patients with partial epilepsy [13,15,154,155]. 

Several models of status epilepticus are also available, most notably pilocarpine and kainic acid models in rodents. These models closely mimic the clinical manifestations of mesial temporal lobe epilepsy in humans. The main characteristics of human temporal lobe epilepsy are: (i) epileptic foci in the limbic system; (ii) an “initial precipitating injury”; (iii) the latent period and (iv) the presence of hippocampal sclerosis. Many of these features are found in the pilocarpine or kainic acid rodent model [61,174,175,176,177]. 

## 9. Conclusions

Despite the huge funding and development of new antiepileptic drugs some 30% of patients are still pharmacoresistant. Currently there is no drug which can prevent epileptogenesis. The treatment of pharmacoresistant patients usually requires polytherapy, therefore these patients are at increased risk of severe side effects and deleterious drug interactions. Hence, there is a need to understand the mechanism of pharmacoresistance and development of new pharmacoresistant animal models which can provide us a new drug with better efficacy and safety profiles than those of older drugs. Any new antiepileptic drug should also be cost effective and display longer duration of action as these properties will improve patient compliance. Due to the heterogeneity and complexity of seizures, a single model cannot be adequate and we need to evaluate a given compound in different animal models before defining its anticonvulsant properties. 
